# Cumingianoside A, a Phyto-Triterpenoid Saponin Inhibits Acquired BRAF Inhibitor Resistant Melanoma Growth via Programmed Cell Death

**DOI:** 10.3389/fphar.2019.00030

**Published:** 2019-01-28

**Authors:** Biljana Cvetanova, Ya-Ching Shen, Lie-Fen Shyur

**Affiliations:** ^1^School of Pharmacy, College of Medicine, National Taiwan University, Taipei, Taiwan; ^2^Agricultural Biotechnology Research Center, Academia Sinica, Taipei, Taiwan; ^3^Graduate Institute of Pharmacognosy, Taipei Medical University, Taipei, Taiwan; ^4^Ph.D. Program in Translational Medicine, College of Medicine, Kaohsiung Medical University, Kaohsiung, Taiwan

**Keywords:** BRAF inhibitor-resistant melanoma, triterpenoid saponin, cumingianoside A, apoptosis, ER stress, autophagy

## Abstract

Mutated proto-oncogene BRAF is a bona fide therapeutic target for melanomas. Regrettably, melanoma acquires resistance to BRAF inhibitors, e.g., vemurafenib (PLX4032) casting doubt on this promising melanoma targeted therapy. In this study, we explored the bioactivity of triterpenoid saponin cumingianoside A (CUMA), isolated from leaves and twigs of *Dysoxylum cumingianum* against PLX4032-resistant BRAF^V 600E^ mutant melanoma A375-R *in vitro* and *in vivo*. Our data show that CUMA treatment inhibited A375-R melanoma cell proliferation in a time- and dose-dependent manner. CUMA also suppressed the activity of CDK1/cyclin B1 complex and led to G_2_/M-phase arrest of A375-R cells. Furthermore, CUMA treatment resulted in induction of apoptosis as shown by the increased activation of caspase 3 and caspase 7, and the proteolytic cleavage of poly(ADP-ribose) polymerase (PARP). We also observed that CUMA induced autophagy-like activity in A375-R cells, as shown by the increased expression of autophagy-related genes and increased formation of autophagosomes. Moreover, we found that CUMA treatment induced ER stress response and co-treatment with an ER stress inhibitor (4-PBA) could attenuate apoptosis induced by CUMA. Importantly, orally administered CUMA as a single agent or in combination with PLX4032 exhibited strong tumor growth inhibition in a PLX4032-resistant A375-R xenograft mouse model, and with little toxicity. This is the first report to explore the anti-tumor activity of CUMA in *vitro* and *in vivo* mechanistically, and our results imply that this triterpenoid saponin may be suitable for development into an anti-melanoma agent.

## Introduction

Melanoma is a significant public health problem with a rapidly increasing rate of incidence worldwide (Siegel et al., 2018). It is the most lethal of the skin cancers with high metastatic potential and is notoriously refractory to conventional therapy (Eggermont et al., 2014; Menzies and Long, 2014). Half of all melanoma patients carry activating mutations in proto-oncogene v-Raf murine sarcoma viral oncogene homolog B (BRAF) that cause constitutive mitogen-activated protein kinase (MAPK) signaling and subsequently, unrestricted melanoma growth (Kumar et al., 2004; Wellbrock and Hurlstone, 2010). Consequently, targeted therapies that specifically inhibit this hyperactive oncogene have revolutionized melanoma treatment. One such example of BRAF^V 600E^ targeted therapy, vemurafenib (PLX4032) has shown unprecedented clinical efficacy (Tsai et al., 2008; Chapman et al., 2011); however, despite its remarkable efficacy, melanoma patients receiving PLX4032 therapy relapse within months. The primary clinical mechanism of acquired melanoma resistance to PLX4032 and other BRAF inhibitors (BRAFi) is the reactivation of MAPK pathway signaling which consequently leads to activation of dysregulated proliferation, aberrant cell cycle progression, and resistance to apoptosis (Lito et al., 2012; Rizos et al., 2014; Van Allen et al., 2014). However, administration of inhibitors of MAPK signaling (e.g., MEK1/2 inhibitors), did not result in any substantial clinical improvement (Tentori et al., 2013; Wagle et al., 2014). Therefore, there is an urgent need for new treatment modalities to treat or sensitize PLX4032-resistant melanoma.

The cell cycle is controlled by checkpoints that consist of cyclin-dependent kinase (CDK)-cyclin complexes which orchestrate progression from one phase to another. Various anticancer agents exert their antiproliferative effects by modulation of the cell cycle regulatory units which leads to growth arrest and consequently apoptosis (Vermeulen et al., 2003; Xu and McArthur, 2016). Various proapoptotic stimuli can result in apoptosis or type I programmed cell death, when typical morphological changes for example, cell shrinkage and condensation, membrane blebbing and adhesion loss can be observed as the result of intracellular proteolytic cascade activation (Elmore, 2007; Ouyang et al., 2012). Autophagy, or type II programmed cell death, is a dynamic catabolic process which sequesters damaged intracellular components into double-membrane vesicles (autophagosomes) and delivers them into the lysosome for degradation (Liu and Debnath, 2016). Endoplasmic reticulum (ER) stress is a condition triggered by impaired ER structure and function, caused by events such as pharmacological stress leading to disruption of ER homeostasis and accumulation of misfolded or unfolded proteins within the ER lumen. The cell senses ER stress by three ER transmembrane sensor proteins, RNA-activated protein kinase–like ER kinase (PERK), activating transcription factor 6 (ATF6), and inositol-requiring enzyme 1 (IRE1) (Schonthal, 2012). By activating the unfolded protein response (UPR) and restoring ER homeostasis, ER stress can be cytoprotective, but when stress is sustained or severe, ER stress can become a cytotoxic signal, mainly by activation of the intrinsic apoptotic pathways (Verfaillie et al., 2013). In BRAF mutant melanoma cells, a non-specific phosphodiesterase inhibitor pentoxifylline activates the ER stress response resulting in the induction of autophagy and apoptosis (Sharma et al., 2016). Although the functional relationship between autophagy and ER stress remains controversial, and both phenomena together determine the fate of the cell (Rashid et al., 2015), emerging evidence suggests that manipulating autophagy and ER stress response in melanoma with acquired resistance to PLX4032 is a promising therapeutic approach (Martin et al., 2015; Cerezo et al., 2016).

Natural products remain an endless frontier for discovery in oncology research due to their novel chemical skeletons, distinct pharmacological activities, and low toxicity profile (Khazir et al., 2014; Newman and Cragg, 2016). For instance, plants of the genus *Dysoxylum* (Meliaceae) have been well documented as a source of structurally diverse chemical constituents with a broad spectrum of pharmacological activities, including antimicrobial, immunomodulatory and anticancer activity (Hu et al., 2011; Jiang et al., 2015). In particular, *Dysoxylum cumingianum* a tree species found in Taiwan, Malaysia and Philippines is a rich source of bioactive triterpenes and triterpenoid saponins (Yoshiki et al., 1992; Toshihiro et al., 1997; Kurimoto et al., 2011). The triterpenoid saponin cumingianoside A was characterized as one of the major constituents in the leaves of *Dysoxylum cumingianum* and was shown to possess anti-cancer activities against various human cell lines including human melanoma cells; however, the detailed anti-cancer mechanism remains unexplored (Yoshiki et al., 1992; Toshihiro et al., 1995).

In this study, we demonstrated the efficacy of CUMA against A375-R, BRAF^V 600E^ mutated human melanoma with acquired resistance to PLX4032 *in vitro* and *in vivo*. Mechanistically CUMA inhibited A375-R growth by inducing G_2_/M phase cell cycle arrest, and ER-stress related apoptosis. Orally administered CUMA significantly and dose-dependently reduced tumor growth in A375-R melanoma xenograft mice that had little or no efficacy to PLX4032. Furthermore, CUMA and PLX4032 combination treatment at reduced administration frequency of either compound/drug exhibited melanoma growth inhibition with negligible mouse weight loss, suggesting that PLX and CUMA combination treatment could be considered as an alternative approach for treating PLX resistant tumors.

## Materials and Methods

### Chemicals and Reagents

Dulbecco’s Modified Eagle Medium (DMEM), Minimum Essential Media (MEM), Roswell Park Memorial Institute 1640 (RPMI 1640), fetal bovine serum (FBS), and the mixture of 100 U/mL penicillin and 100 μg/ml streptomycin were purchased from Invitrogen (Carlsbad, CA, United States). Dimethyl sulfoxide (DMSO), crystal violet, 3-(4,5-dimethylthiazol-2-yl)-2,5-diphenyltetrazolium bromide (MTT), 4′,6-diamidino-2-phenylindole (DAPI), Bafilomycin A1 (Baf A1), 3-methyladenine (3-MA), Chloroquine (CQ), 4-phenylbutiric acid (4-PBA) and Thapsigargin (TG) were supplied by Sigma-Aldrich (St. Louis, MO, United States). PLX4032 was purchased from Selleckchem (Houston, TX, United States). Silica gel was purchased from Merck (Darmstadt, Germany). All chemicals and solvents used in the study were of reagent or high-performance liquid chromatography (HPLC) grade.

### Isolation and Identification of CUMA

**FIGURE 1:**
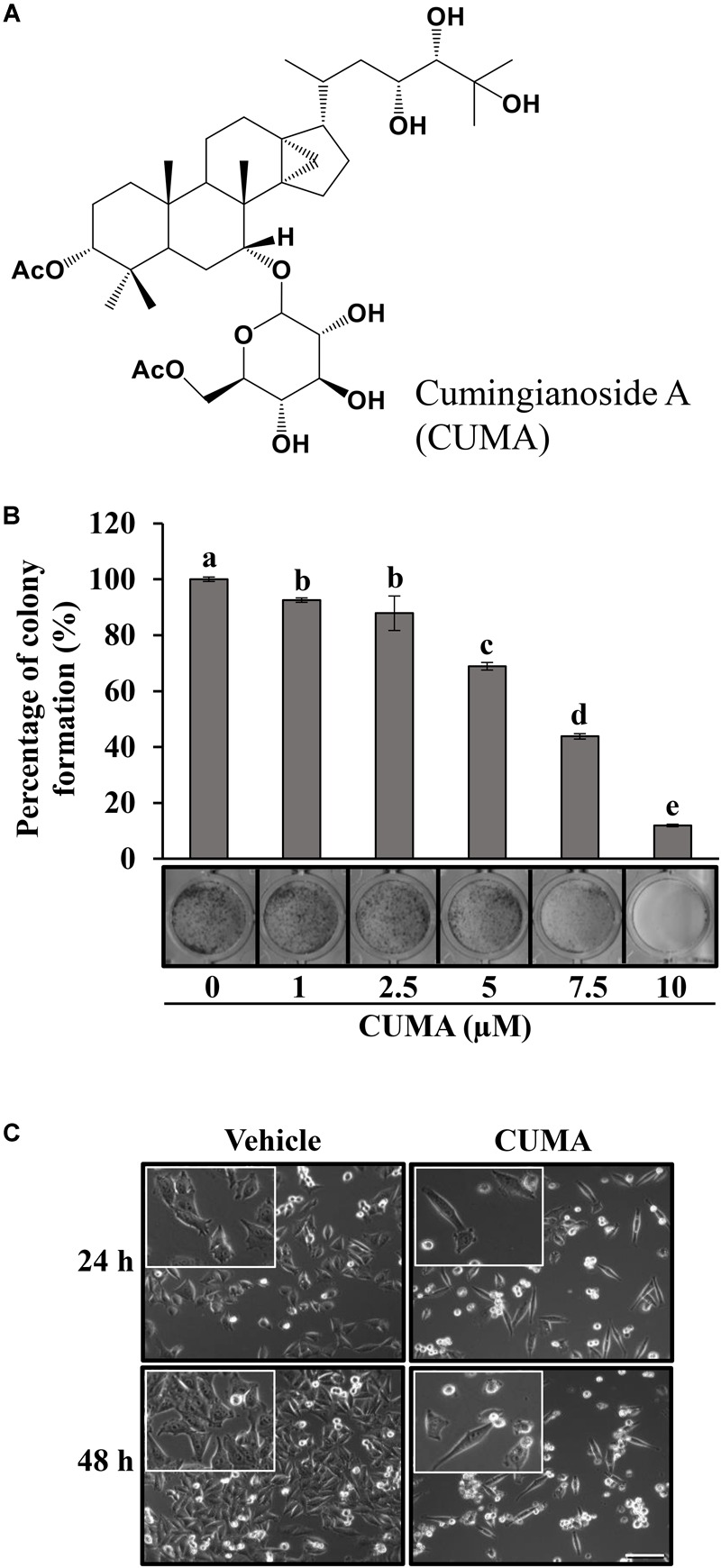
CUMA induces morphological changes in A375-R and inhibits A375-R colony formation. **(A)** Chemical structure of cumingianoside A. **(B)** Colony formation assay of A375-R treated with vehicle and the indicated concentrations of CUMA for 6 days; top, quantification of crystal violet absorbance at 595 nm; bottom, colony stained with crystal violet. Data are mean ± SD, *n* = 3. Different letters indicate significant difference; *P* ≤ 0.05. **(C)** A375-R melanoma cells were treated with 20 μM of CUMA for 24 and 48 h and the morphological changes were recorded by light microscopy (200×, 400× magnification). Scale bar represents 20 μm.

The plant material, *Dysoxylum cumingianum* was collected from Orchid Island, Taiwan, in April 2012 and identified by one of the authors (Y-CS). We established the compound isolation and purification protocols which were modified and simplified from previously published studies (Yoshiki et al., 1992; Kurimoto et al., 2011). Briefly, the acetone extracts from the leaves and twigs of *Dysoxylum cumingianum* were partitioned to yield an EA-fraction which was further subjected to few steps of chromatographic separation using a Sephadex LH-20 column, silica gel column, and in the final step purified by preparative reverse phase high-performance liquid chromatography (Cosmosil 5C18-AR-II column, Nacalai Tesque, Kyoto, Japan) as shown in Supplementary Figure S1, to obtain pentacyclic triterpene glucoside, cumingianoside A (designated CUMA, Figure 1A) with > 95% purity as judged by NMR spectrometry (AVII-500 NMR spectrometer, Brüker, Karlsruhe, Germany). The total mass spectrum of the purified CUMA (rel intensity, positive ion mode: 739.14 [M+H]^+^) determined by electron spray ionization mass spectrometry (Thermo Finnigan LCQ) is shown in Supplementary Figure S2A. The ^13^C NMR and ^1^H spectra of CUMA are shown in Supplementary Figures S2B,C, respectively. The structure was elucidated as 3-*O*-acetyl-3α,7α,23,24,25-pentahydroxy-14,18-cycloapoeuphanyl 7-*O*-β-D-(6′-*O*-acetyl) glucopyranoside, in good agreement with the published data (Yoshiki et al., 1992; Toshihiro et al., 1995).

### Cell Culture

Human melanoma cell lines A375 (ATCC CRL-1619), A2058 (ATCC CRL-11147), SK-MEL-2 (ATCC HTB-68), MeWo (ATCC HTB-65), murine melanoma cell lines B16 (ATCC CRL-6322), B16-F10 (ATCC CRL-6475), and primary epidermal melanocytes (ATCC PCS-200-012) were purchased from the American Type Culture Collection (ATCC, Manassas, VA, United States). A375, A2058 and B16-F10 cells were cultured in DMEM, MeWo, SK-MEL-2, and melanocytes were cultured in MEM, and B16 was cultured in RPMI 1640, supplemented with 10% heat-inactivated fetal bovine serum, 100 U/mL penicillin and 100 μg/mL streptomycin at 37°C in a humidified 5% CO_2_ incubator.

### Cell Viability Assay

Viability studies were carried out by using MTT-based colorimetric assay which quantitatively measures metabolic activity of the viable cells as published elsewhere. Briefly, cells (5 × 10^3^ to 1 × 10^4^ per well) were seeded in 96-well plates and incubated overnight. Test compounds/inhibitors were dissolved in DMSO and diluted in a culture media to a final concentration of 0.5% DMSO. Cells were then treated with various concentrations of test compounds/inhibitors and equal volumes of vehicle (0.5% DMSO) for the indicated times, and further incubated for 3 h with media containing 20 μM MTT reagent. Then, the media was replaced by DMSO and absorbance at 570 nm was measured by ELISA reader. A dose-dependent inhibition curve was used to calculate the IC_50_ (maximal concentration of the tested compound/inhibitor to cause 50% inhibition of the cell viability) values. The data are presented as mean ± SD from four technical repeats and three independent experiments.

### Western Blot Analysis

Western blot analyses were performed as described previously (Chiang et al., 2005). Briefly, total cellular proteins were extracted using radio-immunoprecipitation assay (RIPA) lysis buffer (Santa Cruz Biotechnology, Dallas, TX, United States) containing protease and phosphatase inhibitors. Protein concertation was measured using a colorimetric detergent-compatible protein assay kit (Bio-Rad, Hercules, CA United States) according to the manufacturer’s protocol. Proteins were separated by 10 or 15% SDS-PAGE, and transferred onto a polyvinylidene difluoride membrane (Merck Millipore, Burlington, MA, United States). Blots were blocked in washing buffer (Tris-PBS/0.1% v/v Tween 20) containing 5% w/v skimmed milk for 2 h at room temperature and then incubated with specific antibodies for 16 h at 4°C. After washing, blots were probed with appropriate (anti-rabbit, -mouse or -goat) horseradish peroxidase-conjugated secondary antibodies for 3 h at room temperature. Reactive protein bands were detected using enhanced chemiluminescent detection kit (Thermo Fisher Scientific, Waltham, MA, United States) by exposure to chemiluminescence film, Amersham Hyperfilm ECL (GE Healthcare, Chicago, IL, United States) and quantified by using ImageJ software. Primary antibodies against caspase-7 (cat. #9492), cleaved caspase-7 (cat. #9491), Bim (cat. #2933), phospho-ERK1/2 (cat. #9101), MEK1/2 (cat. #4694), and phospho-MEK1/2 (cat. #9121) were purchased from Cell Signaling Technology; caspase 3 (sc-56053), PARP (sc-7150), Bcl-2 (sc-7382), cyclin B1 (sc-594), CDK1 (sc-54), phospho-CDK1 (sc-12341), Cdc25C (sc-327), p21 (sc-6246), p-Rb (sc-16670), E2F1 (sc-251), ERK (sc-94), MAP LC3B (sc-376404), IRE1α (sc-20790), ATF-6α (sc-166659), PERK (sc-377400) were purchased from Santa Cruz; and β-actin (MAB1501) was supplied from Merck Millipore. Three independent experiments were performed to confirm the reproducibility of the data.

### Animal Studies

The animal experiment to evaluate the therapeutic effect of CUMA against A375-R PLX4032-resistant melanoma was performed according to a protocol approved by the Institutional Animal Care and Utilization Committee (IACUC) of Academia Sinica (Taipei, Taiwan). Six-week-old female NOD/SCID mice bred in the Laboratory Animal Core Facility at the Agricultural Biotechnology Research Center, Academia Sinica were given a distilled water and standard laboratory diet *ad libitum* and kept in a 12 h light/dark cycle at 22 ± 2°C. A375-R cells (3 × 10^6^) were subcutaneously implanted into the right flank of the mice, except for the sham group, and 8 days later when the tumor volume reached 100 mm^3^ the mice were randomly divided into six groups (five mice in the sham group and six mice in every other group) and orally treated with vehicle (5% DMSO and 1% Tween 80 in 0.2 ml of PBS; Tumor control), CUMA (50 and 75 mg/kg body weight; CUMA50 and CUMA75), PLX4032 (50 mg/kg body weight; PLX4032) and CUMA and PLX4032 in combination (CUMA 50 mg/kg body weight and PLX4032 50 mg/kg body weight; CUMA50+PLX4032) once daily in each treatment group except in the combination treatment group in which CUMA and PLX4032 were given alternatively every other day. Tumor dimensions were measured with calipers every 3 days from the beginning of the treatment and the tumor volumes were calculated by formula V = (Length × Width^2^)/2 (Feng et al., 2016). Tumor growth inhibition (TGI) was calculated by the formula TGI (%) = (Vc - Vt)/(Vc - V_0_) × 100, where Vc and Vt represent the mean group tumor volume of the control and treated groups, respectively at the end of the study (day 29) and V_0_ at the initiation of the treatments (day 8). Body weights were measured every 3 days and the percentage of body weight loss was calculated by formula: (B*W* – B*W*_0_)/B*W*_0_ × 100, where BW represents mean body weight of the treated group at day 29 and B*W*_0_ at day 8 (DePinto et al., 2006). At the end of the study, the mice were euthanized and tumors and organs (liver and kidney) were excised and prepared for histological analysis.

### Histology and Immunohistochemistry

The formalin-fixed and paraffin-embedded tumor and organ (liver and kidney) tissues of the test mice were microtome sectioned (5 μm), heat immobilized, deparaffinized in xylene and rehydrated in a graded series of ethanol to water. Organ samples were subjected for hematoxylin and eosin staining (H&E) and tumor samples were subjected for immunohistochemical staining (IHC) and immunofluorescence staining (IF). After antigen retrieval, the following antibodies were used for immunohistochemistry analysis: cleaved caspase-3 (cat. #9661), and cleaved PARP (cat. #5625) from Cell Signaling Technology, VEGF (19003-1-AP) from Proteintech, Ki67 (ab15580) and LC3A/B (ab58610) from Abcam. We used histofine polymer detection system (Nichirei Biosciences, Tokyo, Japan) for detection of the primary antibodies, and 3,3′diaminobenzidine tetrahydrochloride reagent (Leica Biosystems, Wetzlar, Germany) for color development. Hematoxylin (Muto Pure Chemicals, Tokyo, Japan) was used to counterstain the nuclei. For immunofluorescence staining the primary antibody against CD31 (11265-1-AP, Proteintech) was visualized with FITC conjugated secondary antibody (Jackson ImmunoResearch, West Grove, PA, United States). Images were captured on a Zeiss AxioImager Z1 microscope (Munich, Germany) using a Zeiss AxioCam HRc camera and processed using AxioVision Rel.4.9.1 Software.

### Colony Forming Assay

A375-R cells (3 × 10^3^ per well) were seeded in 24-well plates overnight and then treated with the indicated concentrations of CUMA or an equal volume of vehicle for 6 days, the amount of time that is needed for A375-R to form a colony comprising of 50 cells (Franken et al., 2006). Colonies were fixed with methanol, stained with crystal violet and photographed. Inhibition of the colony formation was quantified by measuring the absorbance of crystal violet at 595 nm of the wells containing compound treated cells and comparing with the wells of vehicle treated cells.

### Cell Cycle Analysis

A375-R cells (2 × 10^5^ per well) were seeded overnight in 6-well plates and then treated with different concentrations of CUMA or an equal volume of the vehicle for 12, 24, and 48 h, respectively. Cells were trypsinized, washed with PBS and fixed with 70% ethanol overnight at 4°C. Cells were incubated for 30 min at room temperature in a PBS buffer containing RNase (100 μg/ml), 0.1% triton X-100, and propidium iodide (10 μg/ml) (Lin et al., 2008). Cell cycle distribution was analyzed by flow cytometer Accuri C6 (BD Biosciences, San Jose, CA, United States).

### Apoptosis Assay

A375-R cells (2 × 10^5^ well) were seeded overnight in 6-well plates and then treated with vehicle or CUMA for indicated time. After treatment, cells were harvested, washed with PBS and incubated for 15 min at room temperature in 1× binding buffer containing propidium iodide and FITC-Annexin V as suggested by the manufacturer (BD Pharmingen, San Diego, CA, United States). Apoptotic cells were analyzed by flow cytometer Accuri C6 (BD Biosciences).

### RT qPCR Analysis

To analyze the expression level of autophagy-related genes, total RNA was extracted using Novel Plant Total RNA Mini Kit (Novelgene, Taipei, Taiwan) and reverse transcribed using SuperScript III Reverse Transcriptase reagent kit (Thermo Fisher Scientific, Waltham, MA, United States) as per the manufacturer’s instructions. Quantitative PCR was performed on a 7500 Fast-Real Time PCR system (Applied Biosystems, Grand Island, NY, United States) and data were processed using 7500 Software v.2.3. Delta-delta Ct method was used to quantify the expression of examined genes. The housekeeping gene *GAPDH* was used as an internal control. Primer sequences: *ATG5* (5′-CCAGTTTTGGGCCATCAATC-3′ and 5′-AGTGTGTGCAACTGTCCATCTG-3′), *ATG13* (5′-AGCAGTGGCAATACCCATGA-3′ and 5′-GCATCAAACTCGCGGACATT-3′), *LC3B* (5′-GCAGCATCCAACCAAAATCC-3′ and 5′-TCCGTTCACCAACAGGAAGA-3′), *BECN1* (5′-CTGTGGAAAAGAACCGCAAGA-3′ and 5′-GGGCATAACGCATCTGGTTT-3′), *LAMP-2* (5′-CTGTGCGGTCTTATGCATTG-3′ and 5′-TCATCCCCACAAATGCTTCCT-3′), and GAPDH (5′-TCGGAGTCAACGGATTTGGT-3′ and 5′-ATTTGCCATGGGTGGAATCA-3′) were used.

### Immunofluorescence Cell Staining

A375-R cells (4 × 10^4^ per well) were seeded on coverslips overnight in 24-well plates and then treated with CUMA (20 μM) or equal volume of vehicle for 24 h. Cells were fixed by methanol, blocked with PBS containing 3% bovine serum albumin, and stained with primary antibody (LC3B) and FITC conjugated secondary antibody (Jackson ImmunoResearch, West Grove, PA, United States) with 1:200 dilution. The cell nuclei were stained with DAPI (Shiau et al., 2017). Images were acquired by LSM 780 confocal microscopy (Carl Zeiss AG, Jena, Germany) and LC3B puncta were quantified using ImageJ.

### Statistical Analysis

Quantification of all experimental data are represented as mean ± standard deviation (SD) with the number of experiments indicated in the figure legends. Statistical analysis was conducted by PASW Statistics 18 and significant differences within treatments were determined by one-way ANOVA or Student’s *t*-test. *P* ≤ 0.05 was considered statistically significant.

## Results

### CUMA Inhibits Melanoma Cells Proliferation

To assess the anti-melanoma activity of CUMA *in vitro*, human (A375, A2058, SK-MEL-2, and MeWo) and mouse (B16 and B16F10) melanoma cell lines with different mutational status were treated with CUMA (1–25 μM) for 24 and 48 h and cell viability was determined by MTT assay. CUMA exhibited potent and dose-dependent growth inhibitory effects against all melanoma cell lines harboring BRAF mutation (A375, A2058, B16) or NRAS mutation (SK-MEL-2, B16F10) regardless of whether they were of human or mouse origin as evidenced by the IC_50_ ranging 15.1–22.9 μM and 11.8–15.5 μM at 24 and 48 h treatment, respectively; whereas, for wild-type BRAF and NRAS melanoma cell line (MeWo) IC_50_ of 24.2 μM was observed only after 48 h treatment (Table 1). Importantly, IC_50_ of normal human melanocytes was not observed with a concentration ranging up to 25 μM of tested compound (Table 1).

**Table 1:** Anti-proliferative effect of CUMA against melanoma cell lines with different genetic backgrounds and normal melanocytes determined by MTT assay.

Cell line	Gene mutation	IC_50_ (μM)	
		24 h	48 h
*Normal cell line*			
Melanocyte		-	**-**
*Human cell lines*			
A375	*BRAF*	22.9 ± 1.4	15.3 ± 1.54
A375-R	*BRAF*	15.8 ± 1.93	11.8 ± 0.33
A2058	*BRAF*	15.9 ± 0.86	12.3 ± 0.46
SK-MEL-2	*NRAS*	19.7 ± 2.79	15.5 ± 0.64
MeWo	*no BRAF or NRAS*	-	24.2 ± 0.89
*Mouse cell lines*			
B16	*BRAF*	16.1 ± 3.09	14.6 ± 1.65
B16F10	*NRAS*	15.1 ± 2.47	12.6 ± 1.77

*IC_*50*_, 50% inhibitory concertation of CUMA at indicated treatment time. -, IC_*50*_ not detectable at the measured concentrations up to 25 μM.*

As BRAF inhibitor PLX4032 induced resistance in melanoma patients bearing BRAF^V 600E^ mutation, we also determined the efficacy of CUMA on A375-R, an in-house established BRAF^V 600E^ mutant melanoma cell line with acquired resistance to PLX4032 (Feng et al., 2016). Interestingly A375-R cells showed a higher sensitivity to CUMA as compared to the parental A375 cells with IC_50_ values of 15.8 vs. 22.9 μM at 24 h and 11.8 vs. 15.3 μM at 48 h, respectively (Table 1). We thus focused our investigation on the anti-melanoma activity of CUMA against PLX4032-resistant A375-R melanoma cells, and the underlining mechanisms were examined in the following study.

To analyze the long-term antiproliferative effect of CUMA on the malignant growth of A375-R cells we used a colony-forming assay. As presented in Figure 1B, CUMA treatment for 6 days dose-dependently inhibited colony formation in A375-R cells and with 88% inhibition at 10 μM CUMA. The antiproliferative effect of CUMA was reflected in the decreased confluence of A375-R compared to the vehicle-treated cells at 24 h, as observed by light microscopy (Figure 1C). After 48 h, CUMA treated cells gained typical apoptotic morphological changes including cytoplasmic shrinkage and membrane blebbing (Figure 1C). Therefore, the effect of CUMA on A375-R cell cycle progression and apoptotic cell death were further examined.

### CUMA Induces G_2_/M Phase Cell Cycle Arrest in A375-R Cells

**FIGURE 2:**
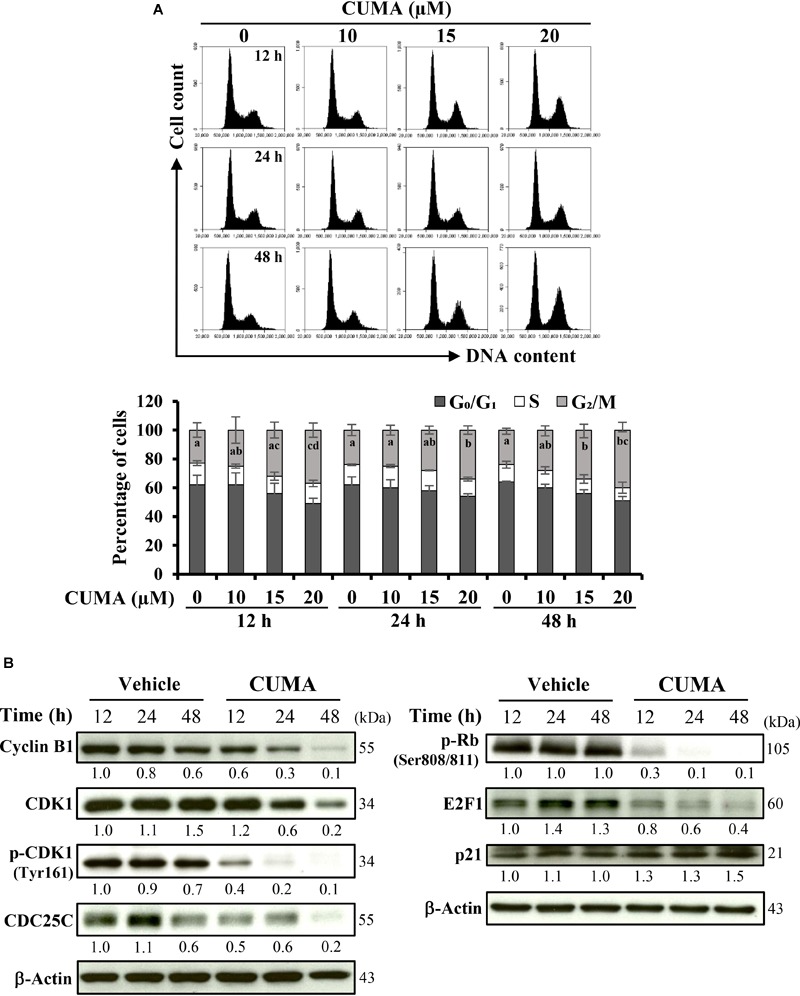
CUMA treatment induces G_2_/M cell cycle arrest in A375-R melanoma cells. **(A)** Cells were treated with vehicle and the indicated concentrations of CUMA for 12, 24, and 48 h, respectively. Top, the cell cycle distribution was measured by PI staining using flow cytometry. Bottom, the percentage of cells in the G_0_/G_1_, S and G_2_/M phase are presented as mean ± SD of three independent experiments. Different letters within the same treatment time group indicate significant difference for the G_2_/M percentage distribution; *P* ≤ 0.05. **(B)** A375-R cells were treated with 20 μM CUMA for the indicated time period and cell cycle-related proteins were detected by western blotting. The expression of the indicated proteins was quantified by densitometry using ImageJ and is presented as fold change vs. vehicle control normalized to the loading control (β-actin).

The regulatory activity of CUMA on the cell cycle of A375-R cells was examined using flow cytometry. As presented in Figure 2A, CUMA induced typical dose-dependent and time-dependent G_2_/M phase arrest. When cells were treated for 48 h with vehicle and increasing concentrations of CUMA (10, 15, 20 μM), the population of cells in G_1_ phase was decreased from 64% in the vehicle group to 56 and 51% (15 and 20 μM CUMA), S phase population was decreased from 12 to 10% and 9% (15 and 20 μM CUMA); whereas in the G_2_/M population it showed an increase from 24 to 34% and 40% (15 μM and 20 μM CUMA) (Figure 2A). At 20 μM CUMA, significant cell cycle arrest was observed earliest at 12 h with 37% of cell population undergoing G_2_/M phase arrest. The longer treatments of 24 and 48 h resulted in comparable effects; 34 and 40%, respectively (Figure 2A). The molecular mechanism for CUMA-induced cell cycle arrest was examined by immunoblotting of proteins involved in the regulation of the G_2_/M phase of the cell cycle. Treatment with 20 μM CUMA induced a time-dependent decrease in the protein expression of cyclin B1 and its associated partner CDK1 (Figure 2B). The phosphorylated form of CDK1^Thr161^ important for its activity was also significantly decreased in the CUMA-treated A375-R cells. One factor that contributes to the decreased phosphorylation of CDK1^Thr161^ by CUMA might be the increased expression of p21, which in addition to its well-known function in regulating G_1_ check point is also known to inhibit G_2_ to M transition by inhibiting CDK1^Thr161^ phosphorylation. Additionally, we observed that the expression level of CDC25C which plays a crucial role in promoting the cell entry into mitosis (Xu and McArthur, 2016) was significantly reduced by CUMA treatment (Figure 2B). Interestingly, we also observed a decrease in phosphorylation of Rb^Ser808/811^ and E2F1 expression (Figure 2B). These data suggest that CUMA inhibited A375-R cell proliferation maybe in part by interrupting the interaction of Rb and E2F1, downregulation of E2F1 transcription factor important for transcription of DNA replication genes, and by inhibition of CDK1/cyclin B1 activity and arrested cells at the G_2_/M boundary.

### CUMA Induces Apoptotic Cell Death in A375-R Cells

**FIGURE 3:**
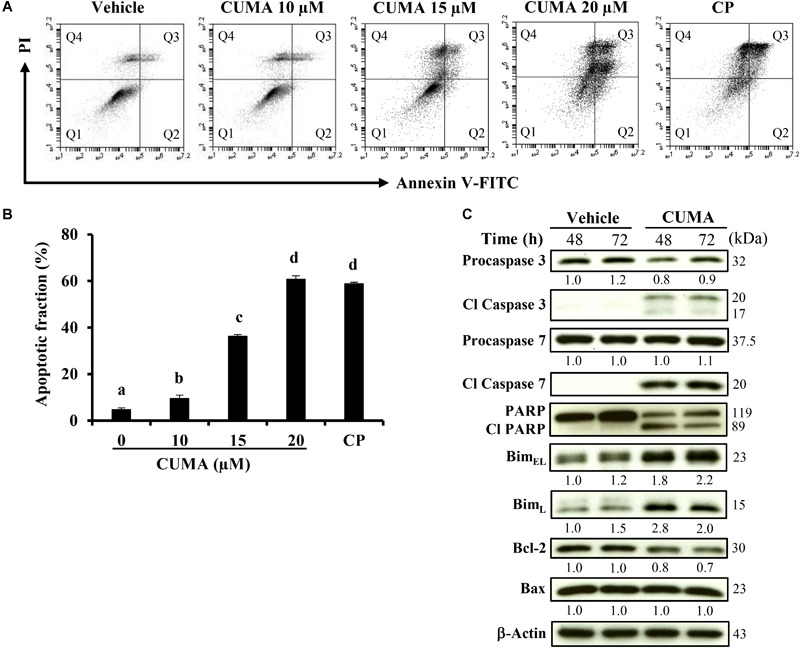
CUMA treatment induces apoptosis in A375-R melanoma cells. **(A)** Cells were treated with vehicle and the indicated concentrations of CUMA for 72 h. Cisplatin (CP) was used as a positive control. Induction of apoptosis was measured by PI/Annexin V double staining using flow cytometry. Q1: live cells, Q2: early apoptotic cells, Q3: late apoptotic cells, Q4: dead cells. **(B)** Apoptotic fraction represents the sum of percentage of the cells in Q2 and Q3. Data are mean ± SD of three independent experiments. Different letters indicate significant difference; *P* ≤ 0.05. **(C)** A375-R cells were treated with 20 μM CUMA for the indicated time period and apoptosis-related proteins were detected by western blotting. The expression of the indicated proteins was quantified by densitometry using ImageJ and is presented as fold change vs. the vehicle control normalized to the loading control (β-actin).

To gain insight into the cell-death mechanism, A375-R cells were treated with the indicated concentrations of CUMA for 72 h, and the apoptotic ratio was analyzed by flow cytometry using Annexin V/PI double staining. The percentage of the apoptotic cell population increased from 5.4% in the vehicle group to 9.5, 36.3, and 60.7% in the 10, 15, and 20 μM CUMA treated cells, respectively (Figure 3A,B). CUMA at 20 μM induced comparable apoptotic death to cisplatin (58.7%) which was used as a bona fide control for induction of apoptosis (Figure 3A,B).

To characterize the molecular mechanism of the apoptotic effect upon CUMA treatment, apoptosis-related factors were examined by western blot analysis. CUMA treatment for 48 and 72 h at 20 μM markedly elevated the levels of the cleaved and activated forms of executor caspase 3 and caspase 7 (Figure 3C). Concordantly, the inactive precursor procaspase 3 was decreased, indicating its activation (Figure 3C). DNA repair enzyme poly ADP-ribose polymerase (PARP) showed decreased activity as observed by the elevated PARP cleavage accompanied by a decrease in the original form (Figure 3C). Several Bcl-2 family proteins regulate the intrinsic mitochondria death pathway and we found that CUMA induced expression of cytotoxic splice variants of the pro-apoptotic proteins Bim_EL_ and Bim_L_ and reduced the expression of anti-apoptotic Bcl-2 but did not change the expression of anti-apoptotic Bax (Figure 3C). Taken together, these results suggest that the mitochondria-dependent pathway might be involved in CUMA-induced A375-R apoptotic cell death.

### CUMA Alone or in Combination With PLX4032 Inhibits Tumor Growth in an A375-R Melanoma With Acquired Resistance to PLX4032 *in vivo*

**FIGURE 4:**
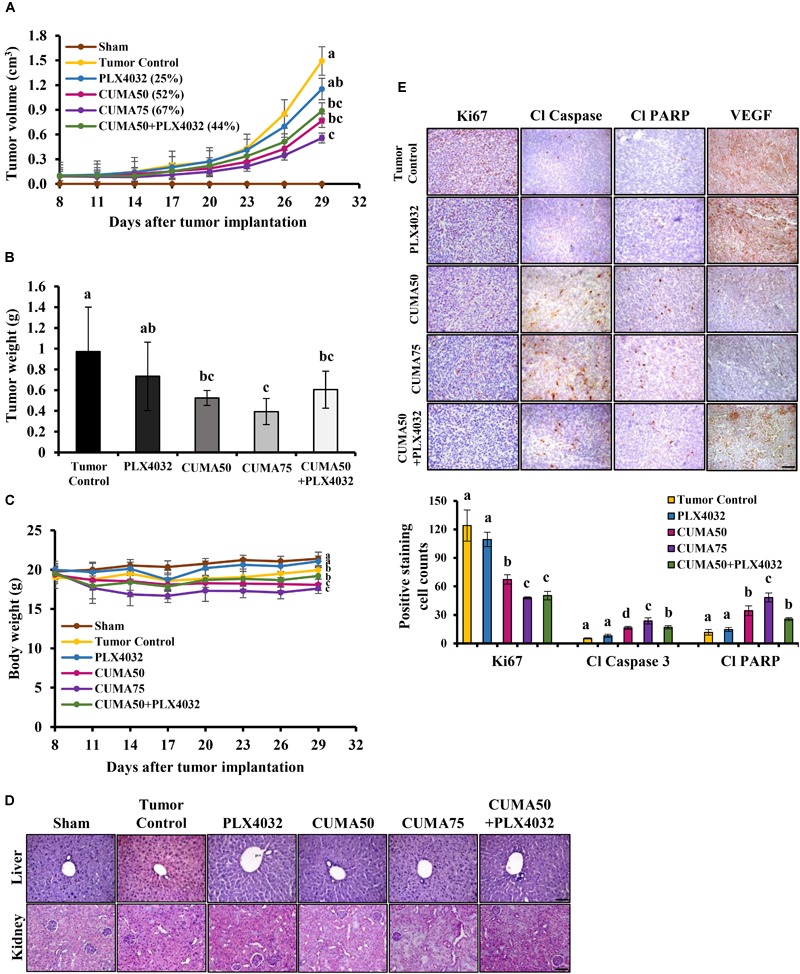
CUMA inhibits BRAF^V 600E^ mutant melanoma with acquired resistance to PLX4032 in a xenograft model. NOD/SCID mice were inoculated with A375-R melanoma cells and when the tumor reached around 100 mm^3^ were orally treated with vehicle (tumor control), CUMA (50 mg/kg/day and 75 mg/kg/day; CUMA50 and CUMA75), PLX4032 (50 mg/kg/day), or CUMA50 and PLX4032 in combination (50 mg/kg/every other day and 50 mg/kg/day; CUMA50+PLX4032). **(A)** Tumor volumes were measured every 3 days and are presented as mean ± SD, *n* = 6 in each treatment group. **(B)** At the end of the study tumors were excised and the weight is presented as mean ± SD, *n* = 6 for each treatment group. **(C)** Mouse body weights were measured every 3 days and presented as mean ± SD, *n* = 5 for sham group, *n* = 6 for every other treatment group. **(D)** Histopathological analysis of the liver and kidney were examined by H&E staining. The integrity of the portal vein and renal glomeruli were examined among the groups. Scale bar represents 50 μm. **(E)** Top, the expression of Ki67, cleaved caspase 3 (Cl Caspase 3), cleaved PARP (Cl PARP) and VEGF (brown staining) in the tumors of different treatment groups were examined by immunohistochemistry. Nuclei were stained blue with hematoxylin. Bottom, quantitative data of the detected Ki67, cleaved caspase 3, cleaved PARP. Data are mean ± SD, *n* = 3. Different letters indicate significant difference with *P* ≤ 0.05. Scale bar represents 50 μm.

We established a A375-R melanoma xenograft model with acquired resistance to PLX4032 to evaluate the potential therapeutic efficacy of CUMA *in vivo* against PLX4032-resistant melanoma growth. The anti-melanoma activity of CUMA and PLX4032 in combination was also evaluated in parallel. The detailed experimental design is presented in Supplementary Figure S3A. Figure Supplementary S3B shows all tumor tissues excised from test mice. PLX4032 treatment (50 mg/kg daily, 22 doses in total) resulted in only minor tumor growth inhibition (TGI) of 25% and tumor weight reduction by 26% and with no statistically significant difference compared to the tumor control group (Figure 4A,B). CUMA low dose (50 mg/kg daily, 22 doses in total) and CUMA high dose (75 mg/kg daily, 22 doses in total) treatment significantly reduced tumor growth by 52% TGI and 67% TGI, and reduced tumor weight by 41 and 59%, respectively, indicating dose-dependent melanoma growth inhibition (Figure 4A,B). Notably, treatment with PLX4032 (50 mg/kg every other day, 11 doses in total) and CUMA (50 mg/kg every other day, 11 doses in total) in combination at reduced administration frequency of either compound resulted in similar effects on test animals to 50 mg/kg CUMA, with 43% tumor weight reduction (Figure 4B). CUMA50 and CUMA75 induced slight body weight loss (6.5 and 10.2%, respectively), while CUMA50+PLX4032 combination treatment caused negligible mouse body weight loss (1.4%) (Figure 4C), suggesting that CUMA and PLX4032 combination treatment with reduced dose frequency of either compound is favorable to general animal health. Further histopathological data showed no notable changes in the structure and morphology in the liver and kidneys among sham, tumor control and treatment groups (Figure 4D), suggesting that CUMA treatment does not cause animal toxicity. The results suggest that the alternative and combinational treatment of PLX with CUMA could be considered as an alternative approach in treating PLX resistant tumors.

*In vivo*, the CUMA anti-tumor effect was observed together with a significant reduction in tumor cell proliferation as detected by Ki67 positive staining regardless of the dose used, and a similar effect was observed in CUMA50+PLX4032 treatment (Figure 4E). PLX4032 treatment showed a higher proliferation rate and Ki67 positive staining similar to the tumor control (Figure 4E). We also observed a marked enhancement in apoptosis as determined by elevated expression of cleaved caspase-3 and increased cleavage of PARP in CUMA-treated animals and higher in CUMA75 compared with the low dose CUMA50 and CUMA50+PLX4032 combination treatment (Figure 4E). Apoptosis markers were slightly increased by PLX4032 and comparable to the tumor control (Figure 4E). The CUMA treatment might impair angiogenesis in the tumors as detected by decreased positive staining of VEGF (Figure 4E) and CD31, a marker representing the presence of endothelial cells (Supplementary Figure S3C) compared to the PLX4032 and tumor control. These data demonstrated that oral administration of CUMA only or PLX and low dose CUMA combination can effectively inhibit the A375-R PLX4032-resistant melanoma growth.

### CUMA Induces ER-Stress Related Apoptosis and Autophagy-Like Activity in A375-R Cells

**FIGURE 5:**
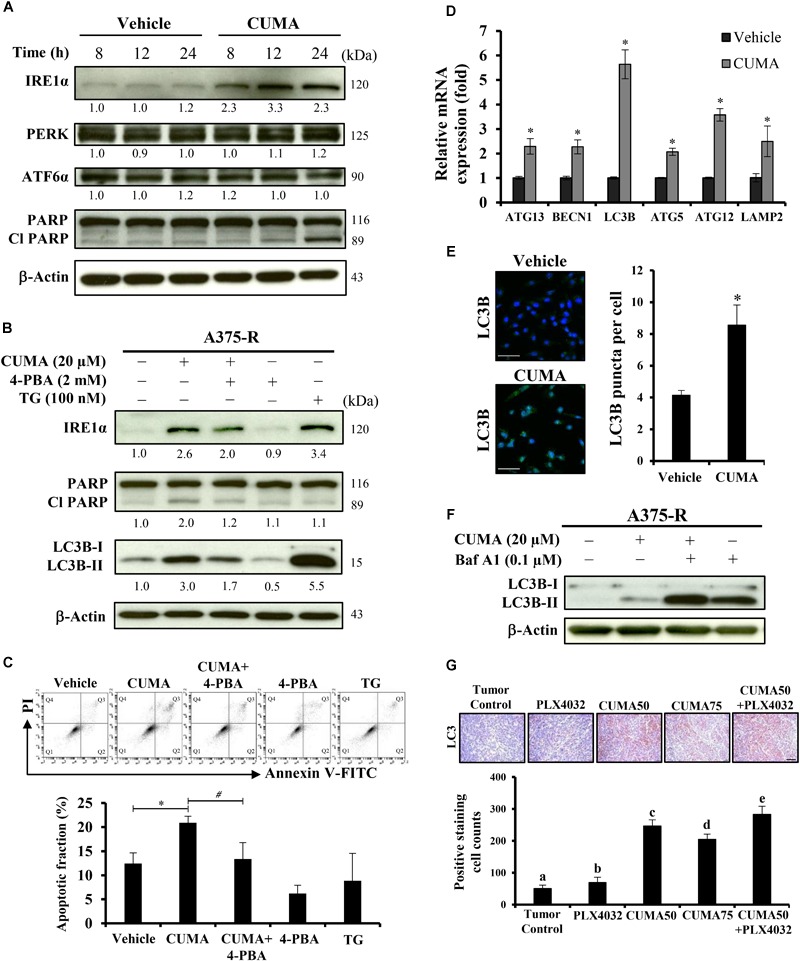
CUMA induces ER-stress related apoptosis and autophagy-like activity in A375-R melanoma cells. **(A)** A375-R cells were treated with 20 μM CUMA for the indicated time period and ER-stress related proteins were detected by western blotting. The expression of the indicated proteins was quantified by densitometry using ImageJ and is presented as fold change vs. the vehicle control normalized to the loading control (β-actin). **(B)** A375-R cells were treated with CUMA (20 μM), 4-PBA (2 mM), CUMA and 4-PBA in combination for 24 h and the protein expression was analyzed by western blotting. TG (100 nM), an ER stress inducer was used as a reference drug in this study. The expression of the indicated proteins was quantified by densitometry using ImageJ and is presented as fold change vs. vehicle control normalized to the loading control (β-actin). **(C)** A375-R cells were exposed to the same treatment conditions as in Figure 5B. Top, the apoptosis was measured by PI/Annexin V double staining using flow cytometry. Q1: live cells, Q2: early apoptotic cells, Q3: late apoptotic cells, Q4: dead cells. Bottom, the apoptotic fraction represents the sum of percentage of the cells in Q2 and Q3. Data are mean ± SD of three independent experiments. ^∗^Significant difference between vehicle control and CUMA treatment; #Significant difference between CUMA and CUMA+4-PBA treatments; *P* ≤ 0.05; (Student’s *t*-test). **(D)** A375-R cells were treated with 20 μM CUMA for 24 h and autophagy related gene expression was examined by qPCR. Data are presented as fold change vs. vehicle control normalized to GAPDH. Data are mean ± SD of three independent experiments. ^∗^Significant difference between vehicle control and CUMA treatment; *P* ≤ 0.05 (Student’s *t*-test). **(E)** A375-R cells were treated with vehicle or 20 μM CUMA for 24 h and LC3B puncta were detected by immunofluorescence staining. Data are presented as mean ± SD number of LC3B puncta (autophagosomes) per cell of at least 100 cells. Scale bar represents 50 μm. **(F)** A375-R cells were treated with CUMA (20 μM), Bafilomycin A1 (0.1 μM), CUMA and Bafilomycin A1 in combination for 2 h and autophagic process was analyzed by measuring the conversion of LC3B-I to LC3B-II. **(G)** The expression of LC3B (brown staining) in tumor tissues was examined by immunohistochemistry. The nucleus was stained blue with hematoxylin. Data are mean ± SD, *n* = 3. Different letters indicate significant difference with *P* ≤ 0.05. Scale bar represents 50 μm.

Induction of prolonged ER stress or suppression of ER stress adaptation mechanisms were proposed as alternative strategies to overcome PLX4032 resistance in melanoma cells (Cerezo et al., 2016). We used immunoblotting to check the expression of PERK, ATF6 and IRE1α in A375-R cells upon CUMA treatment as representative markers of ER stress response. CUMA treatment (20 μM) resulted in the initiation of ER stress, shown by a time-dependent increase in the expression of IRE1α, but with no significant changes in the expression of PERK or ATF6 (Figure 5A). The cleavage form of apoptotic hallmark PARP was increased upon compound treatment (Figure 5A). To investigate the role of CUMA-induced ER stress in cell death we co-treated A375-R cells with CUMA and a chemical chaperone 4-phenylbutiric acid (4-PBA) which attenuates ER stress by promoting protein folding and protein stabilization (Zhang et al., 2013). Thapsigargin (TG) was used as a positive control for ER stress induction (Luan et al., 2015). Interestingly, CUMA-induced IRE1-α and cleavage of PARP were moderately reversed when co-treated with 4-PBA, implying that CUMA-induced ER stress might be partially responsible for the A375-R apoptosis (Figure 5B). To verify the above findings, we performed apoptosis assay under the same treatment conditions as in Figure 5B. When A375-R cells were co-incubated with CUMA and 4-PBA, the percentage of apoptotic cells was reduced from 21% in single CUMA treatment to 13% in the combination which was comparable to the vehicle-treated group (12%) (Figure 5C).

Next, we carried out quantitative real-time polymerase chain reaction (qRT-PCR) analysis to examine the expression of autophagy-related genes including *ATG13, BECN1, ATG5, ATG12, LC3B, LAMP2* in the A375-R cells treated with 20 μM CUMA for 24 h. The data show that all of the tested gene expressions were increased upon CUMA treatment (Figure 5D). To examine the effect of CUMA on the autophagic process, we used two different approaches: fluorescence microscopy to visualize the accumulation of LC3B puncta; and immunoblotting to measure the conversion of LC3B-I to LC3B-II, both of which are indicators of autophagosome formation (Klionsky et al., 2016). We observed that CUMA promoted the accumulation of LC3B puncta (Figure 5E), and when cells were co-treated with CUMA and Bafilomycin A1 (Baf A1), an autophagy inhibitor of autophagosome-lysosome fusion, the level of LC3B-II induced by CUMA was further enhanced (Figure 5F). The same phenomenon of increased LC3 expression was observed in the tumors of mice treated with CUMA, and PLX4032 and CUMA in combination, but to a much lesser degree in PLX4032-treated mice (Figure 5G). To confirm the role autophagy plays in CUMA-induced cell death, we used two autophagy inhibitors, 3-methyladenine (3-MA), which blocks autophagosome formation and chloroquine (CQ), which blocks autophagosome-lysosome fusion (Klionsky et al., 2016). Surprisingly, when A375-R cells were first pretreated for 1 h with either 3-MA (4 mM) or CQ (40 μM) and then additionally treated with CUMA for 24 h, there was no significant alteration in A375-R cell viability compared to the cells treated with CUMA only (Supplementary Figure S4B). Together, these data indicate that CUMA induced autophagy-like activity in A375-R cells; however, whether such effect can be referred to anti-cancer cell proliferation or induction of programmed cell death effect of the compound remains to be further investigated.

## Discussion

The checkpoints orchestrating cell cycle progression have been extensively shown to be dysregulated in late-stage melanomas or melanomas with acquired resistance to BRAFi (Yadav et al., 2014; Azimi et al., 2018). For example, the expression levels of cyclin B1, the regulatory unit of CDK1/cyclin B1 complex important for the transition of G_2_/M is known to be higher in late stage melanomas compared to benign nevi (Georgieva et al., 2001). Thus, one important regulator of CDK1/cyclin B1 complex, CDC25C, is suggested to be a potential oncotarget for melanoma (Capasso et al., 2015). Moreover, hyperphosphorylated Rb and deregulated E2F activation are associated with poor prognosis in malignant melanoma (Singh et al., 2010). In this study, we demonstrated that the triterpene glucoside CUMA significantly suppresses the growth of BRAF^V 600E^ mutant melanoma with acquired resistance to PLX4032 *in vitro*. CUMA effectively inhibited cell cycle progression and inhibited proliferation of A375-R cells, in part through inhibition of the phosphorylation of Rb protein and downregulation of E2F1 transcription factor and inducing G_2_/M cell cycle arrest by affecting expression and/or activation of G_2_/M-phase related proteins CDK1, cyclin B1 and CDC25C (Table 1 and Figure 2). PLX4032 shows prominent inhibition of BRAF^V 600E^ melanoma cell proliferation by arresting the cells at the G_1_-phase of the cell cycle; however, when melanoma cells acquire resistance to the drug, PLX4032 is not able to control the cell proliferation (Smalley et al., 2008; Joseph et al., 2010). We observed a similar effect in the parental A375 (data not shown) and A375-R cells resistant to PLX4032.

During ER stress IRE1α acts as a switch between cell survival and cell death (Li et al., 2012). For example, in hepatoma cells, overexpression of IRE1α inhibited cell growth and repression of IRE1α inhibited ER stress-related apoptosis (Li et al., 2012). In our study, we observed that the increased expression of IRE1α was accompanied by increased cleavage of PARP (Figure 5A) and co-incubation with CUMA and ER stress inhibitor reversed CUMA-induced apoptosis similar to vehicle control, suggesting that A375-R cell apoptosis might be the consequence of CUMA-induced ER stress. We observed similar effects in the parental A375; however, the apoptotic effect was weaker compared to A375-R (Supplementary Figure S5). Further, CUMA treatment increased the levels of the pro-apoptotic molecule Bim which is mainly involved in ER stress-induced apoptosis (Figure 3C) (Puthalakath et al., 2007). We also observed that prolonged incubation with CUMA led to activation of apoptotic hallmarks, caspase 3, caspase 7 and PARP in A375-R and the anti-apoptotic protein Bcl-2 involved in mitochondria intrinsic cell death was decreased (Figure 3C). We have observed that CUMA treatment induced a 1.5-fold increase in ROS levels (data not shown); however, it is not clear whether this phenomenon is associated with CUMA induced ER stress and apoptosis or it may lead to mitochondrial damage in PLX4032-resistant melanoma cells; these points that will need further investigation.

Increasing evidence shows that prolonged ER stress causes not only apoptosis but also another type of programmed cell death, autophagic-cell death (Cerezo et al., 2016). In this study, we observed CUMA induced ER stress-mediated apoptosis in A375-R cells. Further, CUMA treatment increased expression of autophagy-associated genes and marker proteins related to autophagosome formation and accumulation in A375-R cells or in tumor tissues (Figure 5D–G). 4-PBA, a chemical chaperon known to alleviate ER stress reduced CUMA induced conversion of LC3B-I to LC3B-II. These data suggest that CUMA-induced ER-stress might be associated with CUMA induced autophagy-like activity in the A375-R cells. However, autophagy inhibitor 3-MA or CQ pretreatment did not alter the antiproliferative effect of CUMA in A375-R cells (Supplementary Figure S4B) and the exact role of the autophagy-like activity induced by CUMA in the cancer cells needs further investigation.

Reactivation of MEK/ERK signaling is the central mechanism that leads to acquired resistance in BRAF^V 600E^ mutant melanoma, and treatment fails in 50% of melanoma patients (Lito et al., 2012; Rizos et al., 2014). We have indeed observed the paradoxical reactivation of the MEK/ERK pathway in our in-house established A375-R cells (Feng et al., 2016). In this study, although CUMA shows potent inhibition of A375-R cell activity, it does not suppress the protein expression or activation of MEK and ERK in the acquired resistant cells (Supplementary Figure S4A). Further, we found that CUMA also effectively inhibits proliferation of A2058 BRAF^V 600E^ melanoma cells, which are insensitive to PLX4032 despite robust p-ERK inhibition upon PLX4032 treatment (Corcoran et al., 2013). More in-depth investigation to address the potential regulatory role of CUMA on different signaling pathways in the PLX-resistant A375 cells is warranted.

This study is the first to demonstrate the pharmacological activities of CUMA against drug resistant BRAF mutant melanoma. The significance of CUMA is highlighted by the inhibition of the growth of A375-R tumors with acquired resistance to PLX4032 in animals. CUMA significantly inhibited cell proliferation and angiogenesis and induced apoptosis in the tumor tissues (Figure 4E), which is in good agreement with the data obtained from *in vitro* assays for the CUMA inhibitory effect against A375-R cells which were summarized in Supplementary Figure S6. Many natural products with substantial inhibitory activities in cancer cell models display very weak inhibitory activities *in vivo* as a consequence of their unfavorable pharmacokinetics. We observed that CUMA exhibited much less activity in inhibiting A375-R tumor growth by intraperitoneal injection (data not shown), but it showed a potent dose-dependent inhibitory effect when administered by oral gavage. We orally administered a single dose of CUMA at 50 mg/kg, the same dose used in the CUMA+PLX combination treatment, and used LC-MS/MS system to quantify the CUMA level in mouse serum. We did indeed detect the authentic (free) form of CUMA and its respective conjugates to glucuronic acid and sulfate in mouse serum after 24 and 48 h administration retained at concentration of 0.1 ng/ml (data not shown). It may be worth further elucidating the pharmacokinetic mechanism of CUMA in animals to identify other potential bioactive metabolites derived from CUMA.

## Author Contributions

BC, Y-CS, and L-FS conceived and designed the experiments, acquired, analyzed, and interpreted the data. L-FS and Y-CS provided administrative, technical, or material support. BC and L-FS wrote and reviewed the manuscript. L-FS supervised the study.

## Conflict of Interest Statement

The authors declare that the research was conducted in the absence of any commercial or financial relationships that could be construed as a potential conflict of interest.
