# Supplementary material for: Cumingianoside A, a Phyto-Triterpenoid Saponin Inhibits Acquired BRAF Inhibitor Resistant Melanoma Growth via Programmed Cell Death
Source: Front Pharmacol. 2019 Jan 28;10:30. doi: 10.3389/fphar.2019.00030 (PMC6360185; doi:10.3389/fphar.2019.00030)
Supplement: Supplementary file 1 [file Data_Sheet_1.PDF]

Supplementary Information

Cumingianoside A, a phyto-triterpenoid saponin inhibits acquired BRAF inhibitor resistant melanoma growth via programmed cell death

Biljana Cvetanova[1,2], Ya-Ching Shen[1]*, Lie-Fen Shyur[2,3]*

[1]School of Pharmacy, College of Medicine, National Taiwan University, Taipei 10050, Taiwan

[2]Agricultural Biotechnology Research Center, Academia Sinica, Taipei 115, Taiwan

[3]Graduate Institute of Pharmacognosy, Taipei Medical University, Taipei 110, Taiwan

[4]PhD Program in Translational Medicine, College of Medicine, Kaohsiung Medical University, Kaohsiung 807, Taiwan

:** ***Correspondence: *Lie-Fen Shyur, No. 128, Sec. 2, Academia Road, Nankang, Taipei 115, Taiwan; Tel:+886227872102; email: lfshyur@ccvax.sinica.edu.tw; Ya-Ching Shen, No. 33, Linsen S. Rd., Zhongzheng Dist., Taipei 10050, Taiwan; Tel:+886233668773-4; email: ycshen@ntu.edu.tw*

FIGURE S1. Separation scheme of cumingianoside A isolated from leaves and twigs of *Dysoxylum cumingianum*

FIGURE S2. Spectroscopic data of cumingianoside A. (A) Electrospray Ionization Mass Spectrometry (ESI-MS) of cumingianoside A. **(B)** ^{13}C NMR spectra of cumingianoside A in d-pyridine + D_2O recorded by 125 MHz NMR; **(B-1)** Partial ^{13}C NMR spectra of cumingianoside A in d-pyridine + D_2O recorded by 125 MHz NMR. **(C)** ^{1}H NMR spectra of cumingianoside A in d-pyridine + D_2O recorded by 500 MHz NMR; **(C-1; C-2)** Partial ^{1}H NMR spectra of cumingianoside A in d-pyridine + D_2O recorded by 500 MHz NMR

FIGURE S3. Animal study. (A) Detailed experimental design of the A375-R orthotopic xenograft melanoma study and treatment schedule to evaluate the anti-melanoma efficacy of CUMA and CUMA and PLX4032 in combination. The study was conducted for 29 days when mice were euthanized and tumors were dissected for further studies. **(B)** Images of all A375-R tumors dissected from the mice. Scale bar represents 10 mm. **(C)** Top, the expression of CD31 in the tumors of different treatment groups examined by immunofluorescence staining. Nuclei were stained with DAPI. Bottom, quantitative data of the detected CD31 are represented as mean fluorescence intensity relative to the tumor control. Data are mean ± SD, $n = 3$. Different letters indicate significant difference with $P \leq 0.05$. Scale bar represents 50 µm.

Figure S4. CUMA does not suppress MEK/ERK signaling in A375-R cells and inhibition of autophagy by inhibitors did not alter CUMA-induced cytotoxicity in A375-R cells. (A) A375-R cells were treated with CUMA (20 µM) or equal volume of vehicle for the indicated times and proteins were analyzed by western blotting. The expression of the indicated proteins was quantified by densitometry using ImageJ and is presented as fold change vs. vehicle control normalized to the loading control (β-actin). **(B)** Top, A375-R cells were pretreated for 1 h with 3-MA (4 mM) to block autophagosome formation, and then treated with CUMA (20 µM) or vehicle for 24 h. Cell viability was measured by MTT assay. Data are mean ± SD of three independent experiments. ∗: significant difference to the vehicle control; N.S.: not statistically significant to CUMA treatment; $P \leq 0.05$ (Student's *t*-test). Bottom, A375-R cells were pretreated for 1 h with CQ (40 µM) which blocks autophagosome-lysosome fusion, and then treated with CUMA (20 µM) or vehicle for another 24 h. Cell viability was measured by MTT assay. Data are mean ± SD of three independent experiments. ∗: significant difference to the vehicle control; N.S.: not statistically significant to CUMA treatment; $P \leq 0.05$ (Student's *t*-test).

FIGURE S5. CUMA-induced apoptosis can be partially reversed by 4-PBA in parental A375 melanoma cells. (A) A375 cells were treated with CUMA (20 µM), 4-PBA (2 mM), CUMA and 4-PBA in combination for 24 h and the protein expression was analyzed by western blotting. TG (100 nM), an ER stress inducer was used as a reference drug in this study. The expression of the indicated proteins was quantified by densitometry using ImageJ and is presented as fold change vs. vehicle control normalized to the loading control (β-actin). **(B)** A375 cells were exposed to the same treatment conditions as in Figure S5A. Top, the apoptosis was measured by PI/Annexin V double staining using flow cytometry. Q1: live cells, Q2: early apoptotic cells, Q3: late apoptotic cells, Q4: dead cells. Bottom, the apoptotic fraction represents the sum of percentage of the cells in Q2 and Q3. Data are mean ± SD of three independent experiments. ∗: significant difference between vehicle control and CUMA treatment; #: significant difference between CUMA and CUMA+4-PBA treatments; $P \leq 0.05$; (Student's *t*-test); $P \leq 0.05$ (Student's *t*-test).

FIGURE S6. Schematic illustration of the potential mechanism underling anti-melanoma activity of CUMA

Figure S1

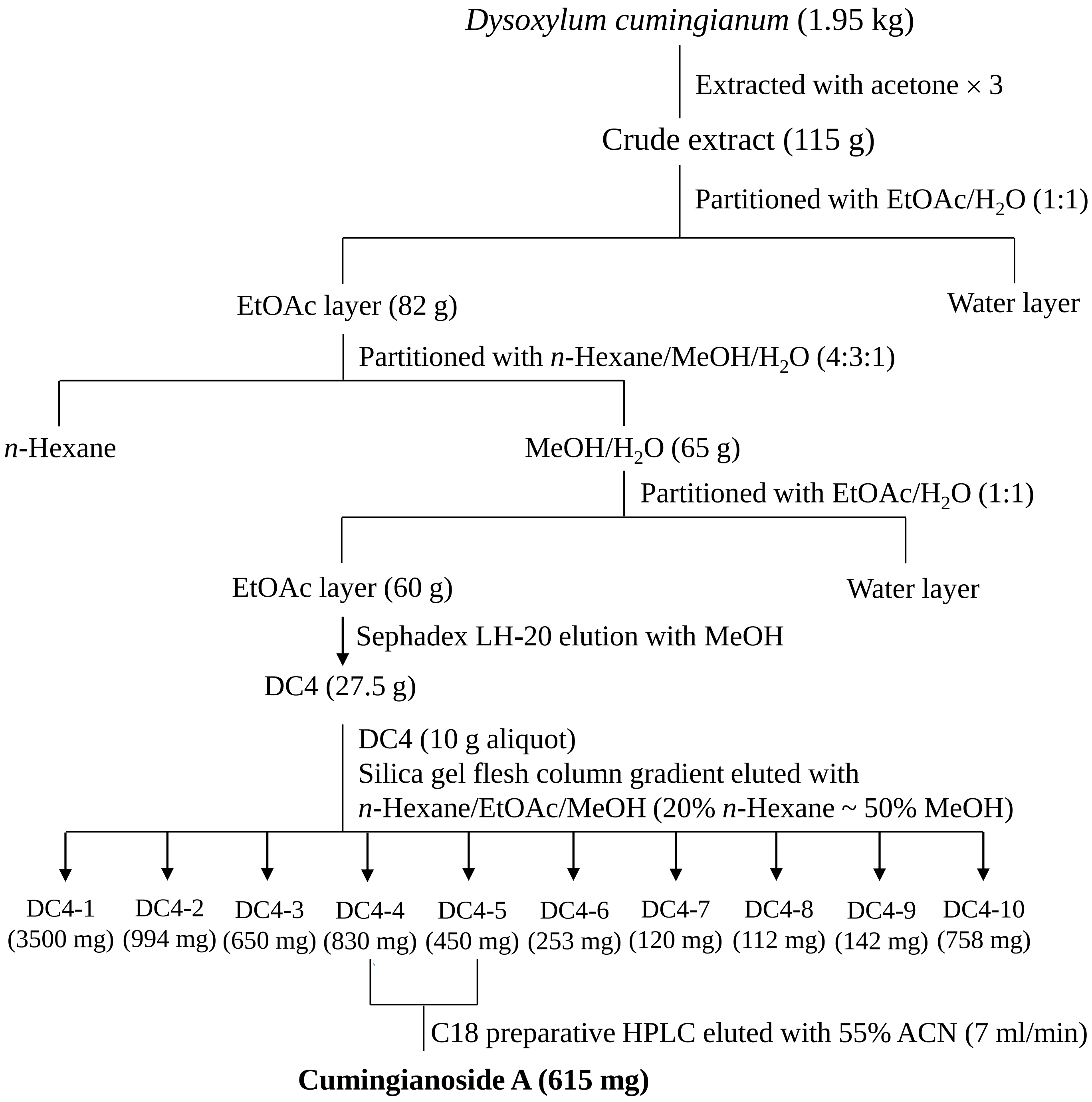
Dysoxylum cumingianum (1.95 kg)
Extracted with acetone × 3
Crude extract (115 g)
Partitioned with EtOAc/H_2O (1:1)
EtOAc layer (82 g)
Water layer
Partitioned with *n*-Hexane/MeOH/H_2O (4:3:1)
n-Hexane
MeOH/H_2O (65 g)
Partitioned with EtOAc/H_2O (1:1)
EtOAc layer (60 g)
Water layer
Sephadex LH-20 elution with MeOH
DC4 (27.5 g)
DC4 (10 g aliquot)
Silica gel flesh column gradient eluted with
n-Hexane/EtOAc/MeOH (20% *n*-Hexane ~ 50% MeOH)
DC4-1 (3500 mg)
DC4-2 (994 mg)
DC4-3 (650 mg)
DC4-4 (830 mg)
DC4-5 (450 mg)
DC4-6 (253 mg)
DC4-7 (120 mg)
DC4-8 (112 mg)
DC4-9 (142 mg)
DC4-10 (758 mg)
C18 preparative HPLC eluted with 55% ACN (7 ml/min)
Cumingianoside A (615 mg)

Figure S2

A

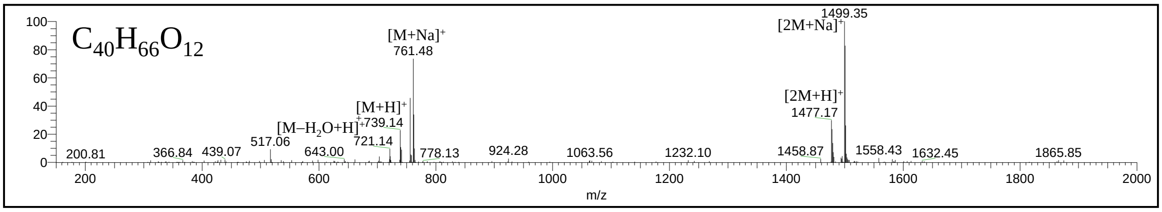

B

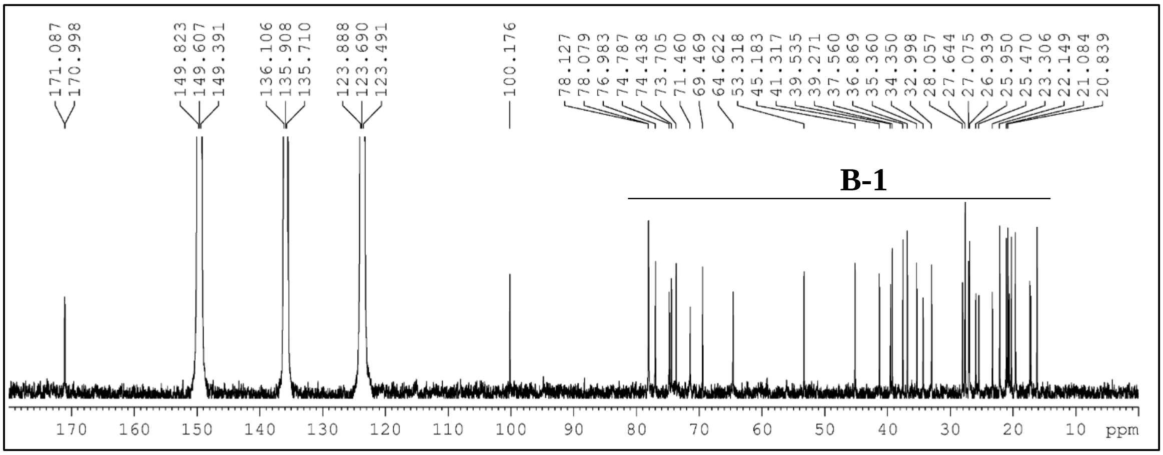

B-1

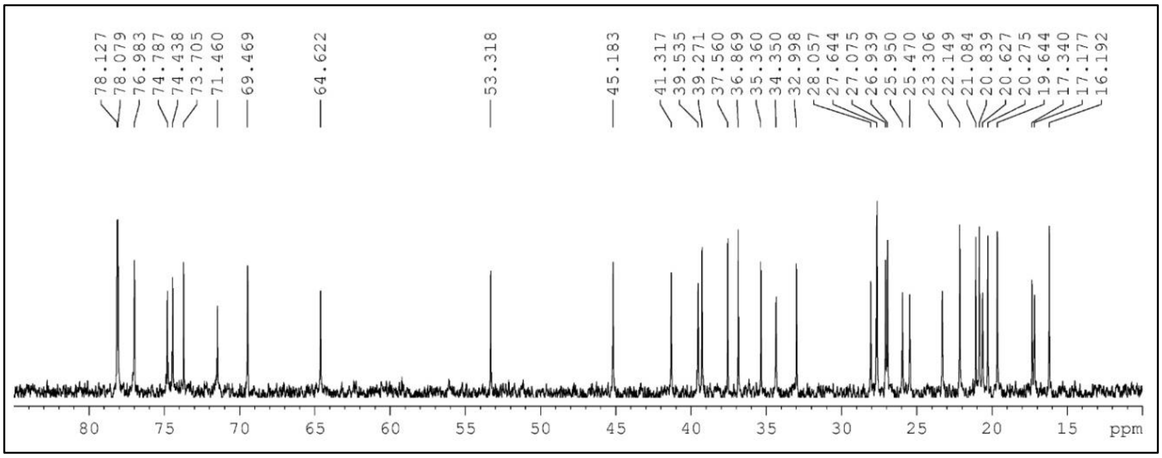

Figure S2

C

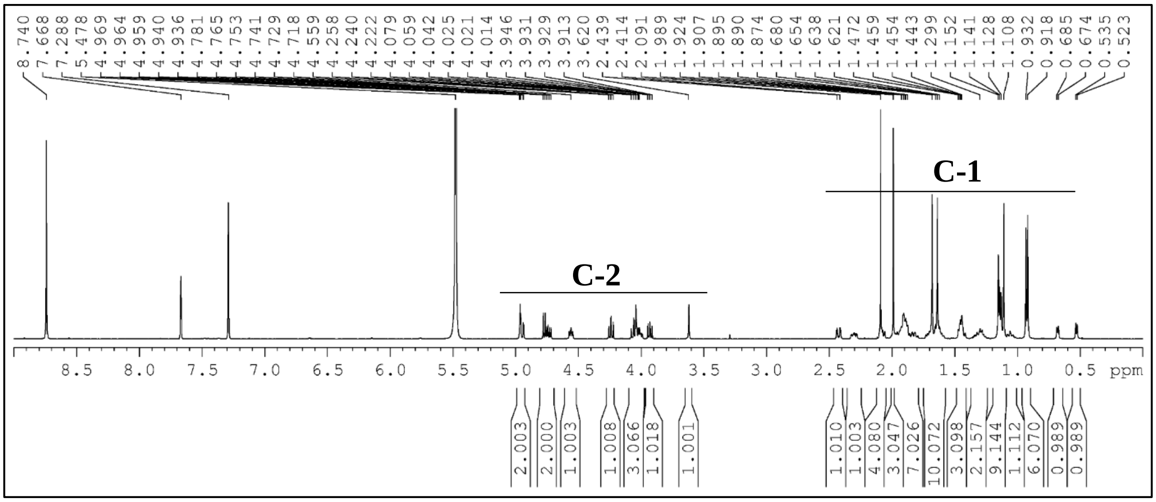

C-1

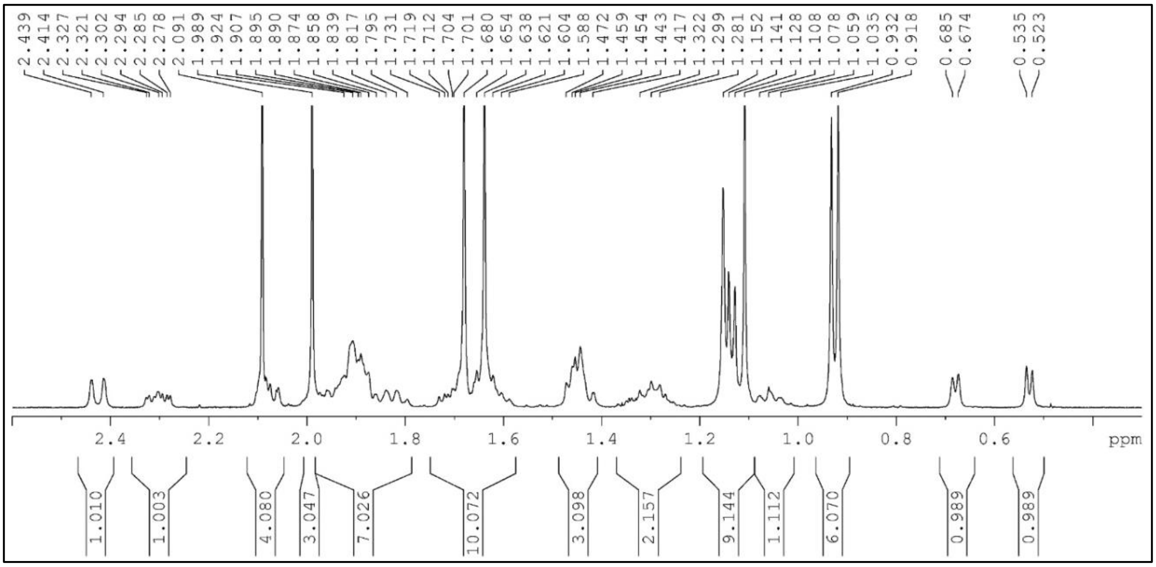

C-2

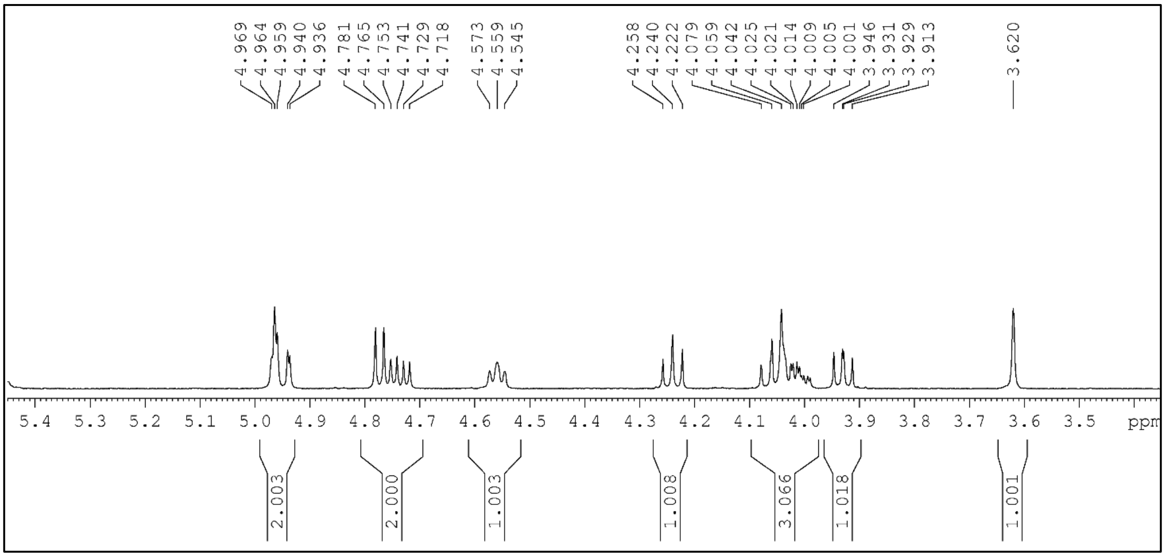

Figure S3

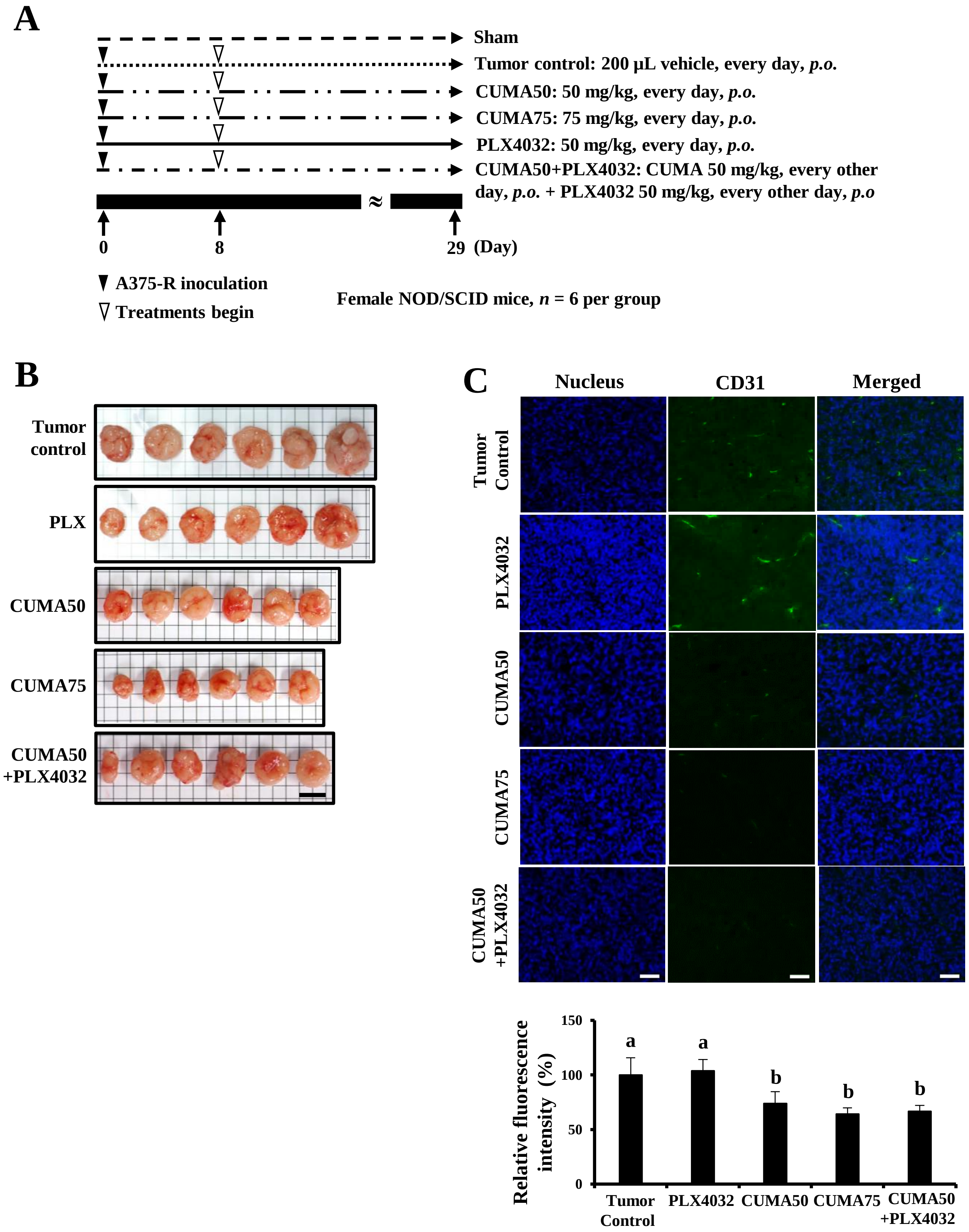

A
Sham
Tumor control: 200 μL vehicle, every day, p.o.
CUMA50: 50 mg/kg, every day, p.o.
CUMA75: 75 mg/kg, every day, p.o.
PLX4032: 50 mg/kg, every day, p.o.
CUMA50+PLX4032: CUMA 50 mg/kg, every other day, p.o. + PLX4032 50 mg/kg, every other day, p.o
0
8
29 (Day)
A375-R inoculation
Treatments begin
Female NOD/SCID mice, n = 6 per group
B
Tumor control
PLX
CUMA50
CUMA75
CUMA50 +PLX4032
C
Nucleus
CD31
Merged
Tumor Control
PLX4032
CUMA50
CUMA75
CUMA50 +PLX4032
Relative fluorescence intensity (%)
150
100
50
0
a
a
b
b
b
Tumor Control
PLX4032
CUMA50
CUMA75
CUMA50 +PLX4032

Figure S4

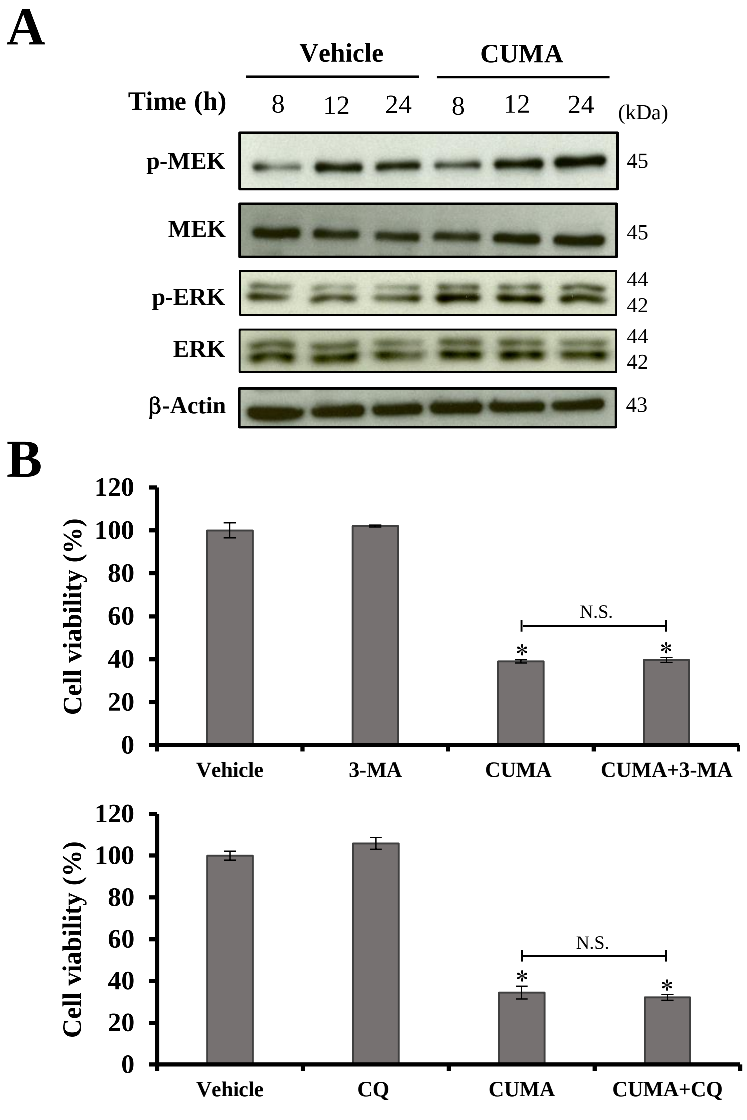
A
Vehicle
CUMA
Time (h)
8
12
24
8
12
24
(kDa)
p-MEK
45
MEK
45
p-ERK
44
42
ERK
44
42
β-Actin
43
B
Cell viability (%)
120
100
80
60
40
20
0
N.S.
*
*
Vehicle
3-MA
CUMA
CUMA+3-MA
Cell viability (%)
120
100
80
60
40
20
0
N.S.
*
*
Vehicle
CQ
CUMA
CUMA+CQ

Figure S5

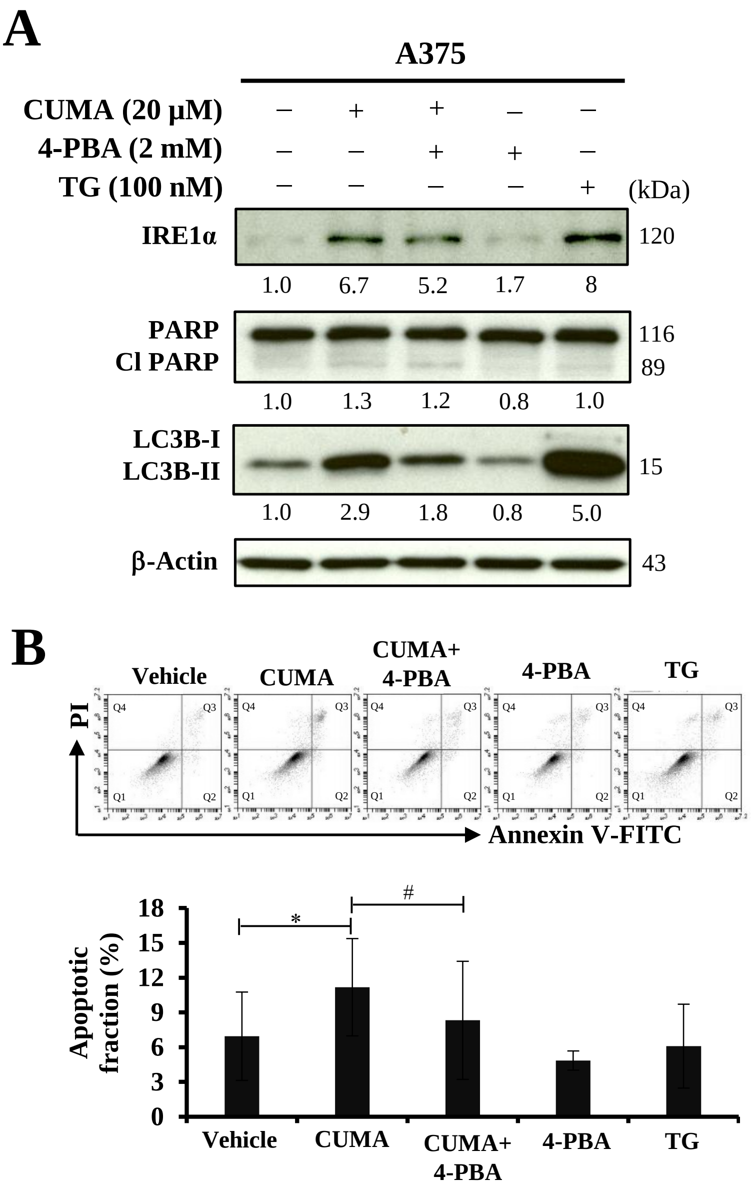
A
A375
CUMA (20 μM) − + + − −
4-PBA (2 mM) − − + + −
TG (100 nM) − − − − +
(kDa)
IRE1α
120
1.0 6.7 5.2 1.7 8
PARP
Cl PARP
116
89
1.0 1.3 1.2 0.8 1.0
LC3B-I
LC3B-II
15
1.0 2.9 1.8 0.8 5.0
β-Actin
43
B
Vehicle
CUMA
CUMA+
4-PBA
4-PBA
TG
PI
Annexin V-FITC
Apoptotic
fraction (%)
18
15
12
9
6
3
0
*
#
Vehicle
CUMA
CUMA+
4-PBA
4-PBA
TG

Figure S6

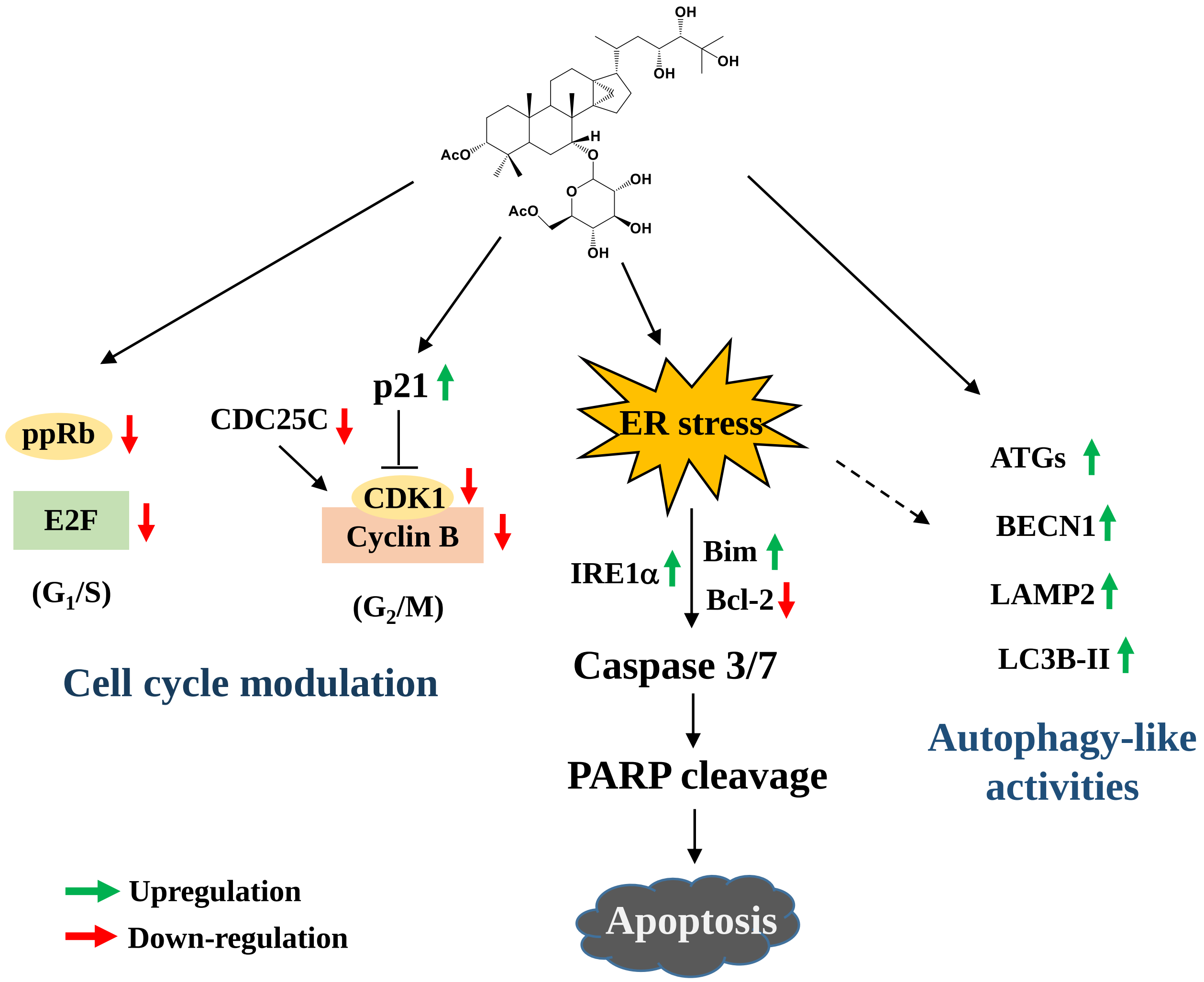

AcO
OH
O
AcO
H
ppRb
E2F
(G_1/S)
CDC25C
p21
CDK1
Cyclin B
(G_2/M)
Cell cycle modulation
ER stress
IRE1α
Bim
Bcl-2
Caspase 3/7
PARP cleavage
Apoptosis
ATGs
BECN1
LAMP2
LC3B-II
Autophagy-like activities
Upregulation
Down-regulation
